# The Utilization of Poly-l-Lactic Acid as a Safe and Reliable Method for Volume Maintenance After Facelift Surgery With Fat Grafting

**DOI:** 10.1093/asjof/ojac014

**Published:** 2022-03-04

**Authors:** Thomas Gerald O’Daniel, Milind D Kachare

**Affiliations:** Department of Surgery (Plastic & Reconstructive Surgery), University of Louisville, Louisville, KY, USA

## Abstract

**Background:**

For age-related volume loss, fat grafting is now recognized as an integral adjunctive procedure with facelift surgery. However, when there is continued and unpredicted volume loss postoperatively, the surgeon has limited options for restoring this lost volume.

**Objectives:**

Poly-l-lactic acid (PLLA) is a proven biostimulator that creates volumetric enhancement. This study is undertaken to demonstrate that PLLA is a safe and efficacious option for maintenance of post-facelift volume loss.

**Methods:**

A retrospective review was conducted to identify all patients who underwent facelift surgery with fat grafting and postoperative PLLA injections from 2010 to 2018 by a single surgeon. Demographic and clinical data were collected and analyzed.

**Results:**

This review identified a total of 241 patients who had undergone a facelift with fat grafting and PLLA injections. Of these, 190 patients were treated with PLLA after facelift and fat grafting, while 51 patients received PLLA injections before their operation. We identified 5 common indications for use of PLLA after facelift surgery and fat grafting. These included unexplained early fat graft loss, significant weight loss in the postoperative period, normal aging process, and patients who had a high perioperative lean body mass. Additionally, PLLA was found to be an effective volumizer for site-specific areas that did not undergo fat grafting during the initial operation. There were no complications reported from the PLLA injections related to nodule formation, papules, or granulomas.

**Conclusions:**

The high degree of variability in the survival of fat grafts with facelift surgery is an accepted reality. PLLA represents a safe and highly effective solution to restore volume loss in patients who have undergone facelifts with fat grafting.

As the human face ages, there is a loss of volume which commonly mimics descent. This particularly occurs in the central facial area as the result of loss of fat within defined compartments and loss of structural skeletal support.^1,2^ Replacement of this central facial volume loss is commonly addressed with nonsurgical means, such as hyaluronic acid (HA) and biostimulators, such as poly-l-lactic acid (PLLA) and calcium hydroxyapatite.^1,3^ Contemporary facelift surgery now recognizes the volume loss that accompanies the peripheral sagging of the face and commonly incorporates fat grafting at the time of facelift surgery to restore central facial volume loss. In fact, a 2015 American Society of Plastic Surgeons (ASPS) questionnaire revealed that 85.2% of respondents use fat grafting during facelifts.^4^

Since the introduction of the modern fat grafting techniques 25 years ago, fat grafting has been touted as the golden standard for volume replacement.^5-7^ The advantages of fat grafting are well known and include an autologous source, no immunogenicity, reduced complications, long-lasting results, relatively low cost, potential of fat integrating into living tissue, and skin improvement among other benefits.^5-7^ Despite the advantages of fat grafting and the extensive research into the factors affecting fat graft retention, there is still a significant degree of variability in graft retention.^2,4,8-11^ Reported volume retention ranges from 20% to 90% and some studies have noted that only patients receiving fat transfers to the cheek were satisfied with the results.^10,12^ In addition to the variability, because fat grafts are biologically active, they may undergo volume changes as a result of the continuum of the aging process, significant weight loss or weight gain.^13^ These factors all have a large impact on the optimal outcome of facial rejuvenation surgery that incorporates fat grafting, which can lead to overcorrection or loss of volume and facial contours.^8,10,14^

Given the accepted variability of fat graft retention, the surgeon has limited options for restoring volume loss, which includes secondary fat injections or injectable fillers. Management of post-surgical volume loss by performing multiple fat grafts is a point of debate as it contains the same unpredictability, may lead to overfilling and is unable to correct smaller surface defects.^7,13^ The senior author’s practice uses HA and PLLA for the management of post-facelift volume loss. HA fillers are used in sensitive areas, such as the perioral, periorbital, or spot-specific midface areas and PLLA is used to restore the structural framework of the midface, lateral face, jawline definition, and temporal hollowing.

PLLA has several advantages as it is biocompatible, biodegradable, and creates collagen stimulation around the injection site.^15,16^ Long-term efficacy results and patient satisfaction in facial volume restoration have been routinely observed with injectable PLLA.^17,18^ For the last decade, the senior author’s practice has incorporated the use of PLLA after facelift and fat grafting for volume maintenance. To demonstrate that PLLA is a safe and efficacious option for maintenance of post-facelift volume loss, a retrospective review was conducted of all patients from 2010 to 2018 who had facelifts with fat grafting and subsequently received PLLA injections.

## METHODS

We performed a single-surgeon retrospective review to identify all patients who underwent a facelift surgery with fat grafting and postoperative PLLA injections over a 9-year period from 2010 to 2018. All patients that fit these criteria were included, while excluding patients that had not received fat grafting or those that received PLLA injections preoperatively or at the time of surgery. The included patients were reviewed for demographics, surgical procedures performed, details of PLLA injection including indications for injection, timing of PLLA related to surgical procedures, number of sessions, volume of PLLA injections, sites injected, and complications related to injections. The ethical principles outlined in the Declaration of Helsinki and Good Clinical Practice were followed, and all patients provided written informed consent for treatment and inclusion to this study.

### Facelift and Fat Injections Technique

There were 2 predominant facelift methods utilized in this time period including a high lamellar sub-superficial musculoaponeurotic system (SMAS) facelift with fat injections for patients who were deemed to have volume depleted-dominant faces and an extended deep plane facelift with fat injections for patients deemed to have accumulation-dominant faces. The fat was prepared with a centrifuge at 1500 rpm for 1 minute. All fat grafting was performed as micro fat grafting utilizing the Tulip harvesting system (Tulip Medical Systems, San Diego, CA) with the 1-mm multi-holed harvest cannula, and infiltration was accomplished with the 0.9-mm Tulip cannula. Injections were performed into the deep compartments, superficial compartments, and subdermal layers to restore the facial shape to the patient’s youthful appearance as was determined from a thorough evaluation of the patient’s younger photographs. Fat injections sites included the midface, periorbital, piriform, and perioral regions including the menton as indicated from preoperative evaluation.

### PLLA Injection Technique

The PLLA (Tradename Sculptra, Galderma, Lausanne, Switzerland) was prepared by reconstituting the product at least 24 hours before use. The senior author’s dilution and anatomical placement are all used in an off-label manner. The PLLA was stored at room temperature. The final volume of the PLLA solution before injection was 7 mL of sterile water and 2 mL of 2% lidocaine with epinephrine for the deep injections and a dilution of 16 mL of sterile water with 2 mL of 2% lidocaine with epinephrine for the deep subdermal injections. Each patient was treated with topical anesthesia (Benzocaine 20%, Lidocaine 8%, Tetracaine 4%, Pentravan Plus Cream [Prospect Compounding Pharmacy, Louisville, KY]) before each injection session. PLLA was injected in the supra-periosteal region to restore volume loss in the temporal fossa (Video 1), midface including the piriform fossa (Video 2) and jawline in the pre-jowl sulcus (Video 3). Deep subcutaneous injections were used in the posterior jawline (Video 4) and the preauricular sulcus. Deep dermal injections were done utilizing a reflex maneuver while tunneling at the subdermal junction and were used to restore dermal lipoatrophy. Post-injection, all patients were instructed to massage the areas of injection for 5 minutes, 5 times per day, for 5 days to ensure an even distribution of the product. Each session utilized a minimum of 1 vial and a maximum of 2 vials of PLLA. The amount of PLLA injected per site was performed in a fashion to create a smooth contour that corrected the areas of defect. We used a minimum of 10 weeks between sessions to evaluate the impact of the previous injections which assisted in precise placement of the subsequent injections to avoid overtreatment.

## RESULTS

From 2010 to 2018, a total of 876 patients underwent facelift surgery. Of these, 788 patients (90%) had simultaneous fat grafting during the operation. Additionally, we identified a total of 792 patients who underwent PLLA injections in this 9-year time frame. Cross referencing these 2 patient populations, we identified 241 patients who had facelift surgery with fat injections and PLLA injections. With further evaluation, 190 (79%) of these 241 patients were treated with PLLA injections after the facelift and fat injections for volume maintenance, while 51 (21%) were treated with PLLA before facelift and fat injection procedures; 233 (97%) were females, while 8 (3%) were male. Fifty-five (23%) of the patients had previous facelifts. The mean age was 62 years (range, 47-87 years). The average time between the facelift with fat grafting operation to the first session of PLLA injections was 14 months and ranged from 6 months to 9 years (Table 1).

**Table 1.** Demographic Analysis of Retrospective Review of the Number of Patients (n) That Underwent Facelifts, Fat Graft Injections, and PLLA Injections

Procedures	n	Mean age (yr)	Female, n (%)	Male, n (%)
Facelifts	876		-	-
Facelifts with fat injections	788	56 (42-85)	-	-
PLLA injections	792	49 (31-85)	-	-
Facelifts with fat injections and PLLA injections	241	-	233 (97)	8 (3)
PLLA injections after facelifts with fat injections	190	62 (47-87)	-	-
PLLA injections before facelifts and fat injections	51	52 (42-59)	-	-

PLLA, poly-l-lactic acid.

Mean ages are shown along with range of age for all patients. The mean time span from facelift with fat injection surgery to the first session for PLLA was on average 17 months (range, 6 months to 9 years).

The total number of vials of PLLA injected to all patients during this time frame was 3786, with an average of 4.78 vials injected per patient. There were 3200 vials utilized in patients who did not have a prior facelift operation, with a mean of 5.3 vials/patient (range, 2-16 vials). The total vials used for patients who had a prior facelift with fat injections were 586, with a mean of 3.2 vials/patient (range, 1-8 vials) (Table 2). All patients reported positive effects from the PLLA injections after reviewing their pre- and post-injection photographs. There was no evidence that advanced age adversely affected patient response to PLLA.

**Table 2.** Number of PLLA Vials Used in Patients From Retrospective Review

Procedures	n
Total PLLA vials injected	3786
PLLA vials injected to patients who only had PLLA injections	3200
PLLA vials injected to patients who had previously done fat injections	586

PLLA, poly-l-lactic acid.

After each injection, the patients were instructed on self-examination for the occurrence of nodule formation. There were no patient-reported complications from the PLLA injections related to nodule formation, papules, or granulomas. At each injection session, the senior author examined the previous injection sites and did not detect nodule formation, papules, or granulomas.

**Figure 1.**
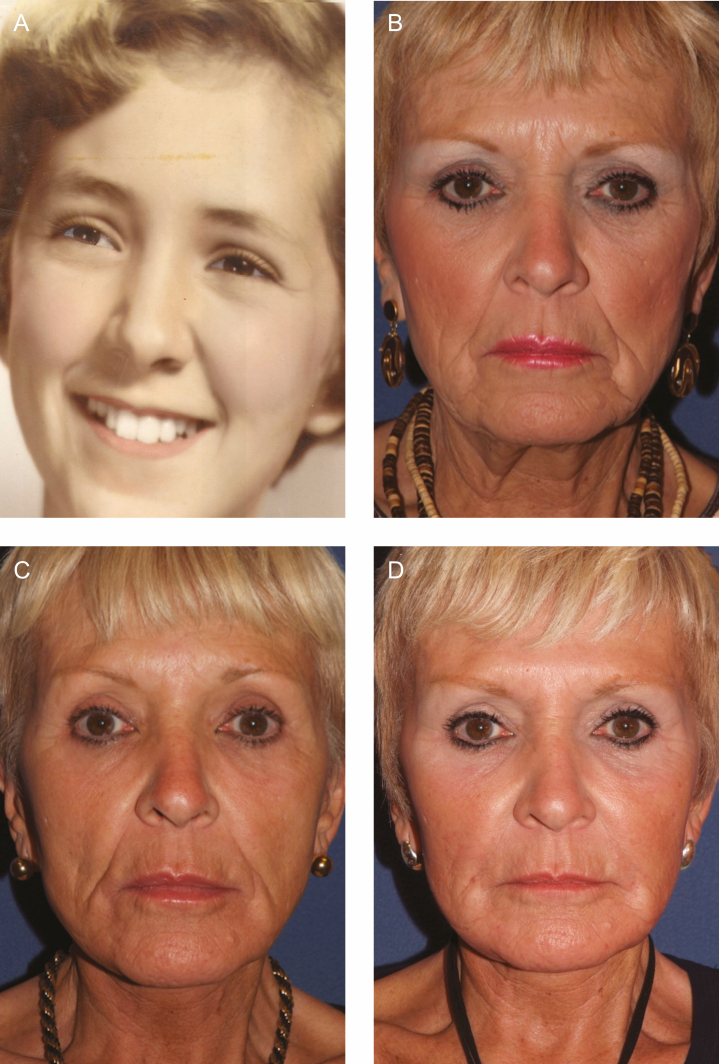
(A) A female patient at 22 years old demonstrating full facial volume. (B) The same patient at 70 years old with significant facial volume loss before facelift with fat grafting and lower lid blepharoplasty. (C) The patient at 71 years old, 1 year postoperative before poly-l-lactic acid (PLLA) injection. (D) The patient at 72 years old, 1 year after the third session of 2 vials/session of PLLA injections to temples, midface including pyriform fossa and pre-jowl sulcus.

**Figure 2.**
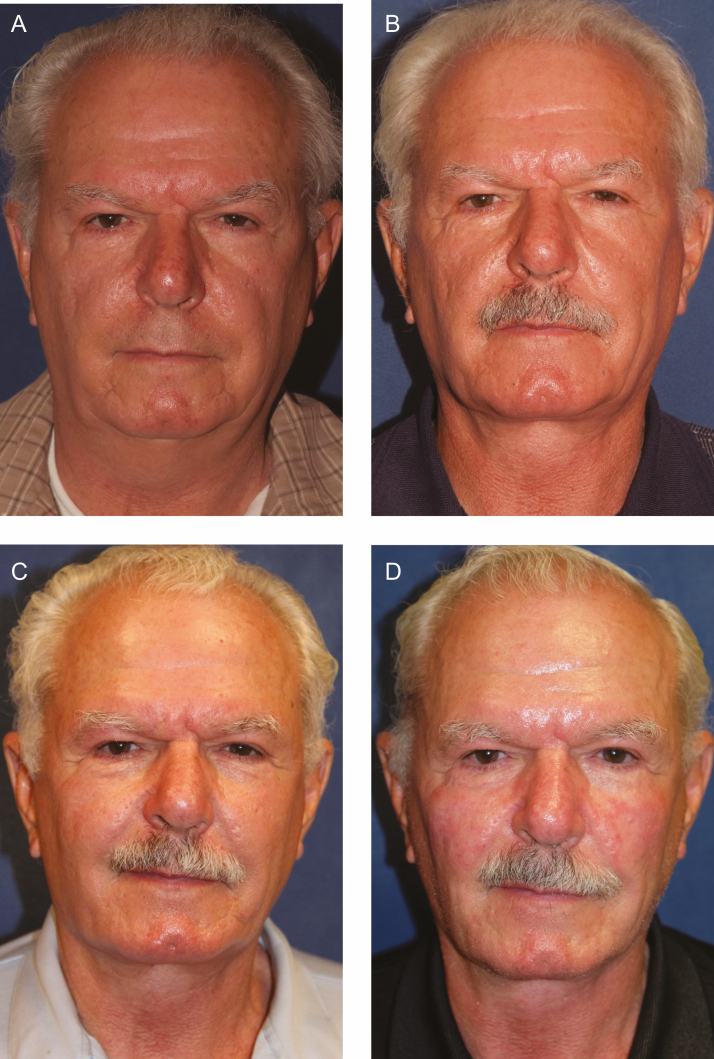
Significant weight loss in the post-facelift and fat injection patient can lead to significant changes in facial volume. (A) This 67-year-old male is shown preoperatively. He had previously undergone a facelift by an unknown surgeon. (B) The patient is 14 months postoperatively from extended deep plane facelift with fat grafting and deep cervicoplasty with reduction of subplatysmal volume including deep fat, anterior digastric muscles, and submandibular glands at the first session of poly-l-lactic acid (PLLA) injection. He exhibits significant loss of facial volume after a 20 lb. weight loss from exercise and dietary changes. (C) The patient is shown 1 year after 3 sessions of PLLA injection, performed every 3 months with 2 vials/session. His weight loss remained stable. Note the significant improvement in the midface volume. (D) The patient is shown at 73 years old, 5 years postoperative, and 4 years after PLLA injections with stable volume.

**Figure 3.**
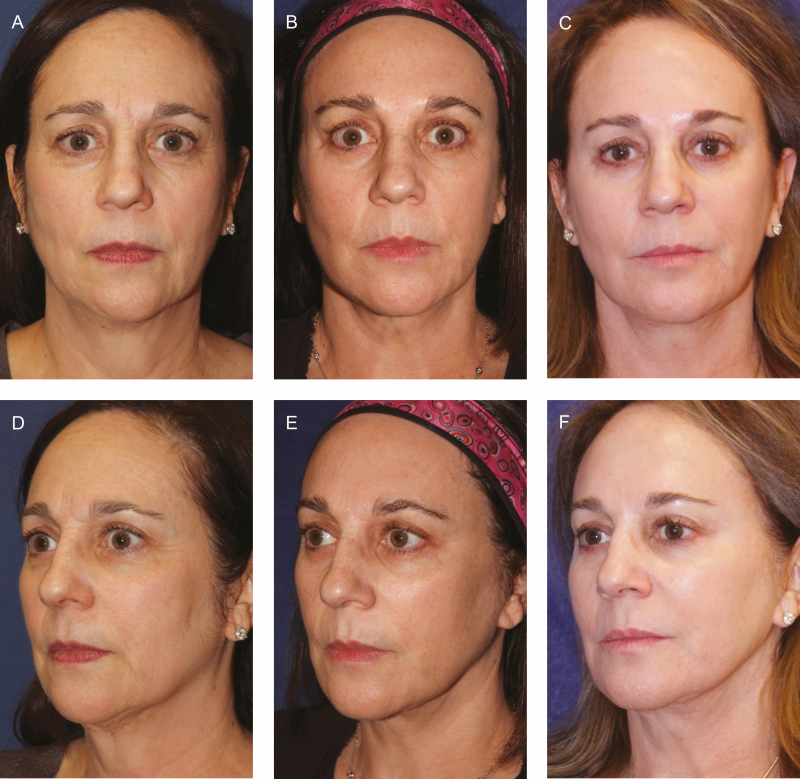
Poly-l-lactic acid (PLLA) is useful for the management of facial volume loss associated with normal aging. A 61-year-old female with preoperative (A) frontal and (D) oblique views. The patient is shown in (B) frontal and (E) oblique views at 64 years old, 2.5 years after facelift, and fat injections before the first session of pan-facial injection of PLLA. The same patient is shown at 70 years old with (C) frontal and (F) oblique view, 9 years after facelift and fat injections, and 6.5 years after having a total of 4 vials of PLLA injected periodically in 4 separate sessions (1 vial/session). During this time, she also had periodic botulinum A injections to the glabella and perioral hyaluronic acid injections.

**Figure 4.**
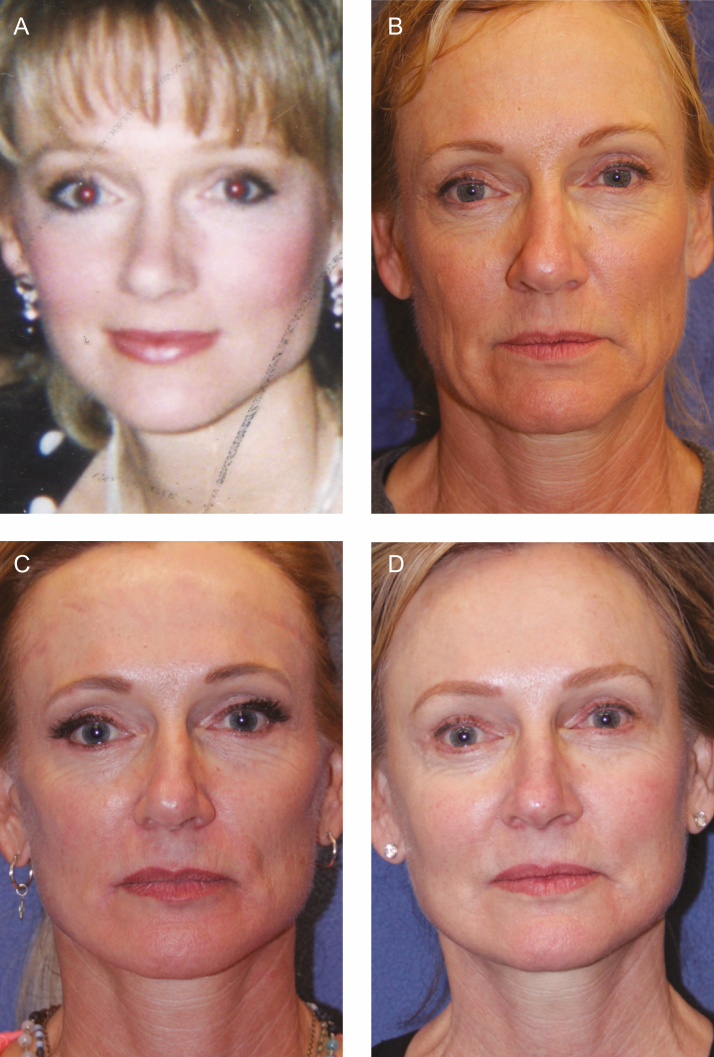
In patients with extreme lean body mass and lack of adequate fat donor sites, we plan for the use of poly-l-lactic acid (PLLA) in the postoperative period to provide central facial volume restoration. (A) A female patient is shown at 24 years old with full facial volume. (B) The same patient, 54 years old, is shown preoperatively. (C) The patient is shown 6 months after facelift and before a session of pan-facial PLLA injection with 2 vials. (D) The patient is shown at 56 years old and 2 years postoperative from facelift and 18 months after PLLA injection.

**Figure 5.**
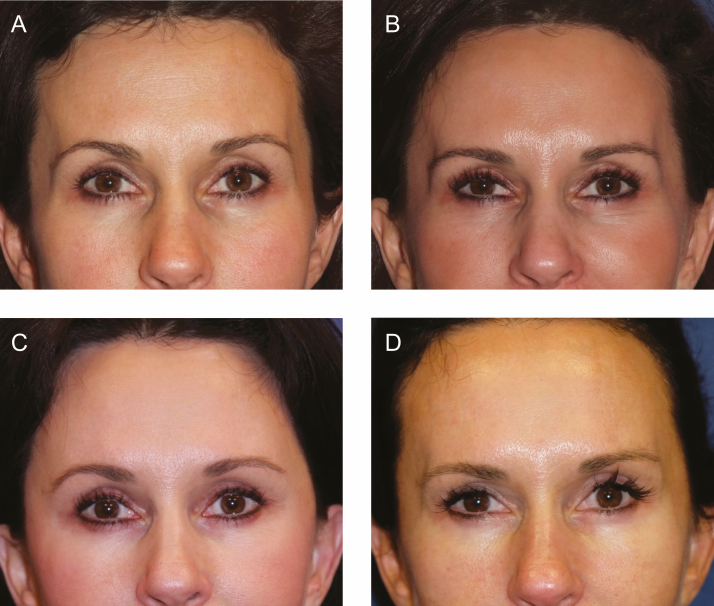
Poly-l-lactic acid (PLLA) can effectively restore temporal volume loss with stable improvement. (A) A 45-year-old female patient is shown preoperatively. (B) The patient is shown 2 years after high superficial musculoaponeurotic system (SMAS) facelift and fat injections with temporal hollowing. (C) The patient is shown 4 years after facelift with fat injections and 2 years after PLLA injections in 2 sessions with 1 vial/session concentrated on temporal hollowing. (D) The patient is shown 7 years after facelift and fat injections and 5 years after PLLA injections.

A review of the patients who had undergone facelifts and fat injections with subsequent PLLA injections identified 5 categories that represented the most common indications for the use of PLLA for volume restoration. These included unexplained early graft loss (Figure 1), significant weight loss in the postoperative period (Figure 2), normal aging volume loss (Figure 3), patients who had a high perioperative lean body mass (Figure 4), and for site-specific treatment in areas that did not undergo fat grafting during the operation (Figure 5).

## Discussion

For more than 5 decades, the aging face has been recognized to be caused by a complex interplay of several major physiological changes that include: reduction in the volume of fat, reduced elasticity, reduced thickness and adherence of skin tissues, increased descent of soft tissues, possible weakening of facial muscles, and the resorption of the craniofacial skeleton.^19^ While our understanding of facial aging has advanced since then, it’s become clear to aesthetic physicians that these age-related changes are a dynamic and multifaceted slew of changes that are best evaluated and treated individually rather than homogenously.^20^ In addition, the changes accompanying the aging face can differ significantly from one individual to another, highlighting the importance of personalized correction strategies addressing the concerns of each patient.^21^

Aging is accompanied by structural changes to the three-dimensional (3D) contours that cause light to reflect differently. These changes in both light and shadows greatly impact the youthful attractiveness of a face. Therefore, restoring 3D contours are essential to restoring a degree of youthfulness. An important recent development has been the integration of volume replacement into the surgical and nonsurgical therapeutic strategies for facial rejuvenation. These strategies must consider the structural characteristics and canvas of the face. Only after careful evaluations, can site-specific treatment strategies be made to achieve natural-looking results.

HA and biostimulators, such as PLLA and calcium hydroxyapatite are the most commonly used nonsurgical materials to augment age-related volume loss of the central face. The most recent American Society of Aesthetic Plastic Surgery survey identified that 1.3 million nonsurgical dermal fillers were used in 2020.^22^ Contemporary facial surgery often incorporates fat grafting during facelifts to further restore facial volume loss. Approximately 85% of plastic surgeons responded that they utilize fat grafting concomitantly with facelift procedures in a 2015 questionnaire to a randomized group of ASPS members.^4^

Both nonsurgical and surgical volume replacement have advantages and limitations related to their usage, efficacy, and longevity. Nonsurgical volume restoration is generally aimed at addressing the changes in the skin and underlying soft tissue that are generally accepted to be the main culprit in age-related loss of facial shape.^1^ In addition, treatments need to address the loss of craniofacial skeletal support of the overlying soft tissues, as this loss contributes significantly to the volume changes which further exacerbates soft tissue descent with aging. Injections of PLLA have been shown to restore skeletal framework and thus restore support of the overlying soft tissue.^1^

**Figure 6.**
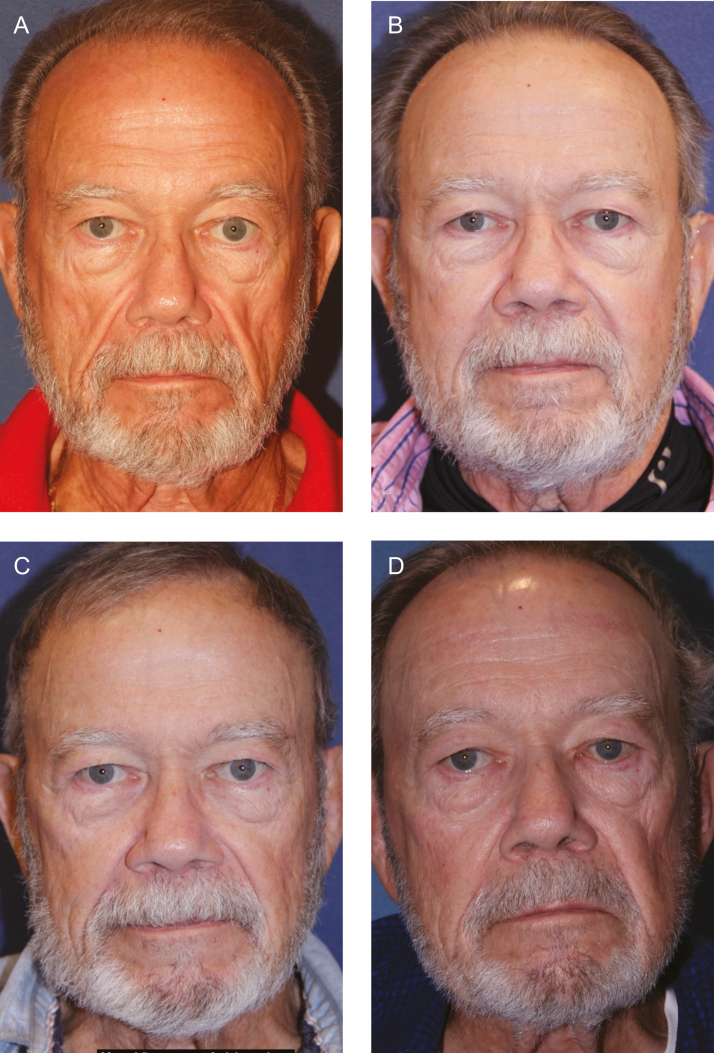
The effects of poly-l-lactic acid (PLLA) volume restoration have been observed for up to 7 years post-injection. (A) A 77-year-old male is shown before 3 sessions of PLLA injection, 2 vials/session, 3 months apart. (B) The same patient 1 year after the third session of PLLA injection. (C) The patient at age 82.5 years old, 4.5 years after PLLA injection. (D) The patient is shown at age 84.5 years old, 7 years after PLLA injection. There is apparent residual volume from the PLLA injections in the central face.

PLLA is a synthetic filler made from an injectable form of PLLA that is biocompatible, immunologically inert, and semi-permanent. It was first approved for use by the FDA for HIV lipoatrophy and subsequently approved for facial rejuvenation procedures requiring the injection of fillers for facial contour deficiencies.^23,24^ PLLA induces neocollagenesis from fibroblasts and does not cause an immediate volumizing effect. Instead, PLLA incites a controlled, mild inflammatory response that leads to its encapsulation and the development of secondary fibroplasia, which in turn enhances volume.^25^ The resulting thickening of the dermal layers has been shown to last for at least 2 years.^26^ In the authors’ experience, positive volumetric results can be seen as long as 7-year post-injection (Figure 6).

In this study, we are suggesting the use of PLLA injections as a nonsurgical option in instances where optimal volume restoration is not achieved or maintained after fat grafting with facelift surgery. The use of PLLA can assist in decreasing the variability in outcomes by reducing the impact of both postoperative fat graft volume loss and age-related structural support loss, which thus contributes to long-lasting results as a maintenance agent.

The continued use of fat grafting for soft tissue augmentation for over a hundred years is a testament of the versatility and safety of fat grafting. Adipose tissue represents a readily available, biocompatible, and inexpensive natural filler that can be easily harvested multiple times from patients with minimal trauma.^27^ Additionally, for more than 20 years, the use of infiltrated autologous fat as a method of filling and supporting the face in 3D lipostructure has been observed to be a safe, efficacious, and lasting method in facial recontouring.^28^ Furthermore, the discovery of stem and regenerative cells in adipose tissue^29^ raised the possibility that autologous fat grafting itself can be leveraged to encourage facial fat tissue restoration and has been demonstrated to lead to improvements in skin texture and pigmentation.^4^

Despite these well-known advantages of fat grafting,^5^ there is a wide variance in reported graft survival from 30% to 80%.^30^ There are several nuisances that have contributed to the significant degrees of variability in the retention of fat grafts, the loss of fat graft volume, and the development of asymmetrical facial contours.^31^ Though we have excellent examples of long-term fat graft stability after facelifts with fat grafting in our practice, the biggest hurdle we still encounter in post-facelift procedures with fat grafting remains our management of the unpredictable degree of fat graft retention and subsequent volume loss that leads to recurring signs of aging as related to loss of structural support.

Several maintenance options are available to mitigate fat graft loss and restore volume loss following facelift and fat grafting, which can lead to an enhancement of results and diminish age-related volume loss. While the second round of fat grafting is an option, these procedures are associated with the inherent risks of a surgical procedure, difficulties in finding suitable donor sites, time for adequate recovery, and the same initial risk of unpredictable fat graft survival. HA injections are the ideal option for restoration of the mobile perioral volume loss; however, the rest of the face typically requires significant volume with stable longevity, such that is difficult to justify the use of HAs as the optimal volume replacement for these areas. In addition, HAs do not work synergistically with fat grafts and have limited capacity to stimulate endogenous collagen production.

**Figure 7.**
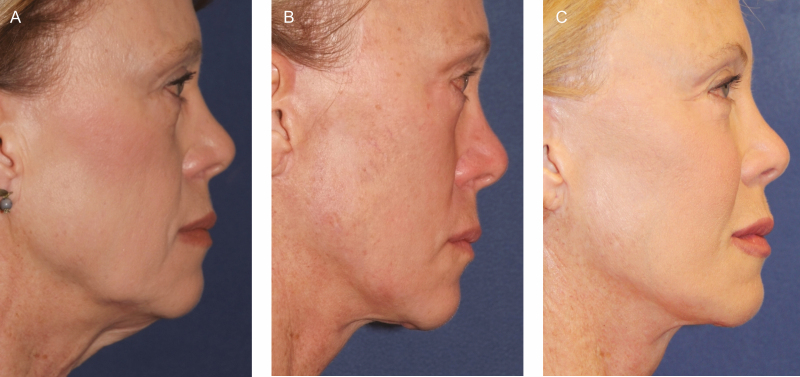
Poly-l-lactic acid (PLLA) is used to restore the facial framework of the central face. (A) A 64-year-old female patient is shown preoperatively. (B) The same patient is shown 1 year postoperatively after high sub-superficial musculoaponeurotic system (SMAS) facelift, 4 lid blepharoplasty, and fat injections with minimal maintenance of the central facial fat injections, before the first of 3 sessions of PLLA injections, 2 vials per session. (C) The patient is 66 years old and 1 year after the third session of PLLA injection.

Alternatively, PLLA injections may avoid many of the previously mentioned complications and challenges encountered with subsequent rounds of fat grafting and/or HA injections. The injection of PLLA is a minimally invasive procedure that acts synergistically with fat grafts to optimize post-surgical volume. The injection of PLLA in the post-facelift and fat graft patient has been shown to augment facial volume by improving the facial framework as seen in this figure showing central facial volume restoration (Figure 7).^32^

In reviewing our outcomes from facial rejuvenation procedures with fat grafting and given the accepted rate of fat grafting loss as high as 80%, we do not feel that fat graft loss is necessarily a complication. More importantly, we as surgeons, as well as patients, must acknowledge the impermanence of any procedure and that natural aging leads to a continual loss of volume irrespective of facial rejuvenation procedures. In our cohort of patients who subsequently underwent PLLA injections after fat grafting, we identified various possible causes for the postoperative volume loss. In some cases, excessive volume loss can be due to inadequate graft survival without any discernible cause for the fat grafting loss (Figure 1). Patients often experience postoperative weight loss that leads to changes in lean body mass that can lead to a reduction in facial fat and contribute to the volume loss of fat grafts (Figure 2). Additionally, patients with low body fat percentages generally have less available donor sites for fat grafting and require alternative volumizers, which are often determined before the surgical procedure (Figure 3). Because of these observations, our preoperative consultation includes a thorough discussion of these postoperative scenarios in which fat graft volume is lost or inadequate and offer the option of PLLA injections. This preoperative discussion has led to wide acceptance of the use of PLLA for variable fat graft retention, which is used in 25% of our post-facelift and fat injection procedures.

When we compare the surgical patients receiving PLLA for volume restoration to those receiving PLLA as the sole volume replacement, we find that less PLLA volume and fewer injection sessions were necessary to reach the patient’s expected endpoint. Less volume requirement was most likely because of the impact of the previous grafting, as well as tissue repositioning from the sub-SMAS facelift (Table 2). The most obvious reason for less PLLA injected in postoperative patients is because of the varied survival of the fat graft, mitigating the need for higher volumes of PLLA. However, an interesting postulation that cannot be supported by current research is that the PLLA somehow interacts synergistically with the grafted fat cells improving volume.

Patients who have previously undergone facelift and fat grafts and desire to keep the youthful, rejuvenated appearance afforded by the procedure will require continual maintenance as aging leads to continual fat and bone loss. We identified a group of patients in which the fat injections had adequate survival and PLLA was used to maintain the optimal postoperative appearance over a long period of time (Figure 4). In the authors’ experience, the addition of PLLA injections is an ideal maintenance tool for volume and has a significant impact on patient retention. Making the patient aware of the impermanence of all procedures and products keeps the client open minded about future procedures. The patient will ideally return to the operating surgeon’s facility for future treatments, including PLLA.

Common complications from PLLA injection include bruising and post-injection discomfort, while rare complications include the development of papules and granulomas, which is known to have an estimated incidence between 0.1% and 1%.^33^ Through our experience, we had no patient reported occurrence of nodules, papules, or granulomas in patients who had a facelift, fat injections, and PLLA injections. This was confirmed by physical examination by the senior author at each injection session.

It has been mentioned in various forums that PLLA can create an inflammatory condition that makes it difficult for the surgeon to elevate the various tissue planes required during facelift surgery. In our review of the operative records of patients who underwent PLLA injection before facelift with fat grafting procedures, we did not experience an impediment in any aspect of the surgical procedure. The authors did experience areas where the PLLA had created a thickened and firm tissue response, particularly in the lateral fixed SMAS layer and in the sub-SMAS plane over the zygomaticus muscle. These tissue changes, however, did not prevent safe and effective elevation of the SMAS in the course of the facelift. In certain instances, the lateral fixed SMAS was obviously thickened, which enhanced the effectiveness of the SMAS suspension (Video 5). Therefore, previous PLLA injections should not be considered a hindrance to future a sub-SMAS facelift.

The limitations of the study include the retrospective nature of the study as well as a single-surgeon experience. Also, we did not have the capacity to perform objective volumetric assessment of the fat graft retention in patients who had or did not have postoperative PLLA injections. Most of our patients who had facelift with fat grafting procedures did not undergo PLLA injections postoperatively, but this does not imply adequate fat graft survival. PLLA injections were also performed often in the early preoperative time span. Additionally, a recent study by Cohen^9^ showed a “rebound” of early fat graft volume loss at 18 months which suggests a normal cycle of fat graft integration may take up to 18 months to see the final results. This rebound effect could account for some of the positive impact we see from the injections of PLLA. Finally, there is an absence of objective patient satisfaction data in regard to either the impact of fat graft volume loss and/or the necessity to perform injection of PLLA postoperatively.

## CONCLUSIONs

Facelift with fat grafting has become commonplace with all various types of face lifting techniques. There is a high degree of variability in the survival of the fat grafted. PLLA represents a highly effective solution to mitigate volume loss in patients who have undergone facelifts and fat grafts. PLLA can be safely utilized to restore volumes to satisfactorily levels and therefore represents a cost-effective option. Patients are amenable to the use of PLLA for volume restoration for its numerous advantages.
